# Exploring the use of telemedicine to expand addiction and recovery resources in Great Plains Tribal Communities

**DOI:** 10.1111/jrh.70113

**Published:** 2026-01-14

**Authors:** Michael C. Harding, Anna C. Kihlstrom, Allison Kelliher, Carmen Rosa, Frankie B. Kropp, T. John Winhusen, Donald K. Warne

**Affiliations:** ^1^ Johns Hopkins Bloomberg School of Public Health Baltimore Maryland USA; ^2^ University of North Dakota Grand Forks North Dakota USA; ^3^ Center for the Clinical Trials Network National Institute on Drug Abuse Bethesda Maryland USA; ^4^ University of Cincinnati College of Medicine Cincinnati Ohio USA

**Keywords:** access to care, drug abuse, health disparities, qualitative research, technology

## Abstract

**Purpose:**

Addressing substance use disorders remains a high priority for many Indigenous communities. Opioid misuse and deaths related to overdose have been increasing sharply in American Indian/Alaska Native populations. Medications for opioid use disorder (MOUD) remain difficult to access in Great Plains Tribal Communities due to the paucity of treatment providers, among other factors. The present study explores the perceived barriers and facilitators to using telehealth to promote access to MOUD and recovery resources in Great Plains Tribal Communities.

**Methods:**

This study employed qualitative methods to review policy considerations for using telemedicine to provide buprenorphine. We obtained qualitative data from 5 interviews with 8 total key informants (62.5% women, 25% with tribal affiliations) with local administrators, health care providers, and policymakers. Their responses were transcribed and coded with NVivo software.

**Findings:**

After coding and analysis, 6 themes emerged: current access, acceptability in Tribal Communities, facilitators, barriers, payment considerations, and policies that support tele‐MOUD. Participant responses—though specific to Great Plains Tribal Communities—mirrored other recommendations on telemedicine and substance use disorder services such as federal support of reciprocity of state licenses, permanent codification of the regulatory changes enacted during the COVID‐19 public health emergency, increased funding for innovative delivery of services, and considerations of privacy; the need for culture‐ and trauma‐informed providers was also noted.

**Conclusions:**

Telemedicine for the provision of MOUD appears, from this qualitative analysis, to be a feasible way to expand access to care for opioid use disorder in Great Plains Tribal Communities.

## BACKGROUND

Although opioid use and misuse in the United States is as old as the nation itself, the Harrison Narcotic Control Act of 1914 was the first regulatory framework for controlled substances, making it unlawful for any person to “produce, import, manufacture, compound, deal in, dispense, sell, distribute, or give away” coca or opium without proper registration and tax compliance.^1^ It also required that opioids only be available by prescription and discouraged maintenance therapy for chronic use. In 1970, the Comprehensive Drug Abuse Prevention and Control Act, commonly known as the Controlled Substance Act (CSA), replaced the Harrison Act. The CSA created the federal schedule—which stratified controlled substances based on acceptable medical use, the potential for abuse/addiction, and harmfulness—and reinforced prescription requirements, namely that a controlled substance “must be issued by a practitioner in the usual course of their practice and be issued for a legitimate medical purpose.”^2^ The Drug Addiction Treatment Act of 2000 (DATA 2000) broadened “legitimate medical purpose” to include prescribing controlled substances that are US Food and Drug Administration (FDA)‐approved for the purpose of addiction treatment.^3^ Of note, the 2 opioid agonists approved for treatment of opioid use disorder, methadone and buprenorphine, are schedule II and III, respectively.

In the early 2000s, awareness of a new opioid epidemic was building. Overprescription of long‐term opioid analgesics and, more specifically, predatory prescribing practices, underpinned the growing numbers of individuals with opioid‐use disorder and opioid overdose deaths. The Ryan Haight Online Pharmacy Consumer Protection Act of 2008 was designed to reduce the harm caused by the unethical dispensing practices of certain online pharmacies. The heart of the legislation mandates that “No controlled substance that is a prescription drug as determined under the Federal Food, Drug, and Cosmetic Act may be delivered, distributed, or dispensed by means of the Internet without a valid prescription.” The paragraph that follows underscores that a “valid prescription” is predicated upon “a medical evaluation that is conducted with the patient *in the physical presence* of the practitioner” (emphasis added).^4^ Notable exceptions to the in‐person exam exist under the Ryan Haight Act, including the provision of services during a Secretary of Health & Human Services‐designated public health emergency and the provision of services by Indian Health Service (IHS) designated Internet Eligible Controlled Substance Providers (IECSPs).

Under Part 3, Chapter 38 of the *Indian Health Manual*, the IHS Director is the responsible party charged with designating eligible prescribers as IECSPs. Providers wishing to become an IECSP must hold an active and unrestricted State medical license, local medical staff privileges, and an active Drug Enforcement Administration (DEA) registration to prescribe controlled substances. Until recently, this included an active Drug Addiction Treatment Act of 2000 (DATA 2000) waiver to conduct maintenance and detoxification treatment using the controlled substances approved for treatment of Opioid Use Disorder (OUD). By policy, designated IECSPs serve remote areas, that is, geographic Primary Care Health Professional Shortage Area (HPSA) criteria (HPSA Score > 15—or 75% unmet need) or any other areas determined by the IHS Director to be sufficiently remote related to geography and/or access to services.^5^

In a 2018 announcement from then Indian Health Service Chief Medical Officer, Dr. Michael Toedt explained, “It is sometimes difficult in rural and remote locations to access a provider with the necessary training and approval to prescribe buprenorphine in an outpatient or office‐based setting. This policy enables IHS, Tribal, and Urban Indian Organization health care providers to apply to be designated by IHS as Internet Eligible Controlled Substance Providers, allowing them to prescribe controlled substances for Medication Assisted Treatment through telemedicine.”^6^, ^7^

During the COVID‐19 public health emergency, regulatory changes expanded the use of telemedicine for prescribing buprenorphine, removing some of the bureaucratic hurdles that previously limited accessibility. Secretary Azar declared COVID‐19 a public health emergency on January 31, 2020. While the state of public health emergency remained in effect, DEA‐registered practitioners were permitted to issue prescriptions for controlled substances (including buprenorphine) to patients for whom they had not conducted an in‐person medical evaluation.^8^ Further rollback of regulations included the elimination of the DATA waiver to prescribe buprenorphine‐containing products.^9^ After the conclusion of the public health emergency in 2023, the DEA, together with the Department of Health and Human Services (HHS), extended the telemedicine exceptions for buprenorphine prescribing through December 31, 2025.^10^

Substance misuse has long been identified as an issue of concern among American Indian/Alaska Native (AI/AN) populations. Over the past 2 decades, the age‐adjusted annual mortality rate among AI/AN has increased steadily^11^ before spiking sharply from 2018 to 2021.^12^, ^13^ As of 2022, the age‐adjusted rate of drug overdose death was higher for AI/AN patients (65.2 per 100,000) than for any other race/ethnicity category.^14^ These disparities are underpinned by historical and contemporary trauma, including genocide, land dispossession, dissolution of family and community ties, and cultural erasure.^15^, ^16^, ^17^

Given the recent expansion of telemedicine for the treatment of opioid use disorder, the provision of addiction and recovery services to Tribal communities with unmet needs remains an area of great importance. The present study, supported by the National Drug Abuse Treatment Clinical Trials Network (CTN‐0129), explores the perceived barriers and facilitators to using telehealth to promote access to addiction and recovery resources in Great Plains Tribal Communities.

## METHODS

Investigators used a qualitative approach to identify tele‐MOUD policies, best practices, and perceived barriers and facilitators for tele‐MOUD. Tele‐MOUD in this study was understood to mean remote visits via audio/visual platform between a patient with opioid use disorder and a provider able to prescribe medications for opioid use disorder (MOUD). Study participants responded to a 9‐question, modified version of the Community Readiness Assessment tool (see Supplement A)^18^ to obtain perspectives on the acceptability and feasibility of tele‐MOUD. Four individual interviews and 1 focus group were completed via an internet audiovisual platform. A total of 8 key informants were identified through snowball sampling and included local administrators, behavioral health providers, or policy makers from the Indian Health Service or the Substance Abuse and Mental Health Services Administration. Participants signed informed consent agreements and were compensated with a $20 Visa Gift Card. All relevant ethical safeguards have been met in relation to patient or subject protection. The study was approved by the university's Institutional Review Board.

The qualitative data analysis for this study involved a multistep process. Initially, the 5 recordings were transcribed using Otter transcription software. Each transcript was meticulously reviewed against the original recordings to ensure accuracy and fidelity. Following the transcription process, the finalized transcripts were imported into NVivo version 14.24.0 (QSR International) for further analysis.

Within NVivo, a combination of deductive and inductive coding methods was employed. Deductive coding was applied to align with the predetermined themes derived from the interview questions, encompassing 6 main thematic areas. Subsequently, inductive coding techniques were utilized to identify additional emergent concepts within each predefined category. Five iterative rounds of coding were conducted, during which codes were continuously refined, renamed, or eliminated based on their relevance and accuracy in representing the data. This comprehensive coding process ensured a thorough exploration of the interview transcripts and facilitated the extraction of meaningful insights relevant to the study objectives.

The Great Plains Area of the Indian Health Service includes Tribal communities located in North Dakota, South Dakota, Nebraska, and Iowa. Many of these communities are classified as medically underserved according to the Health Resources and Services Administration criteria, and all but one of our partner sites were situated in nonadjacent, nonmetro counties with an urban population under 5,000, which corresponds to a Rural Urban Continuum Code (RUCC) of 9 based on the 2023 RUCC classification.^19^

## RESULTS

**FIGURE 1:**
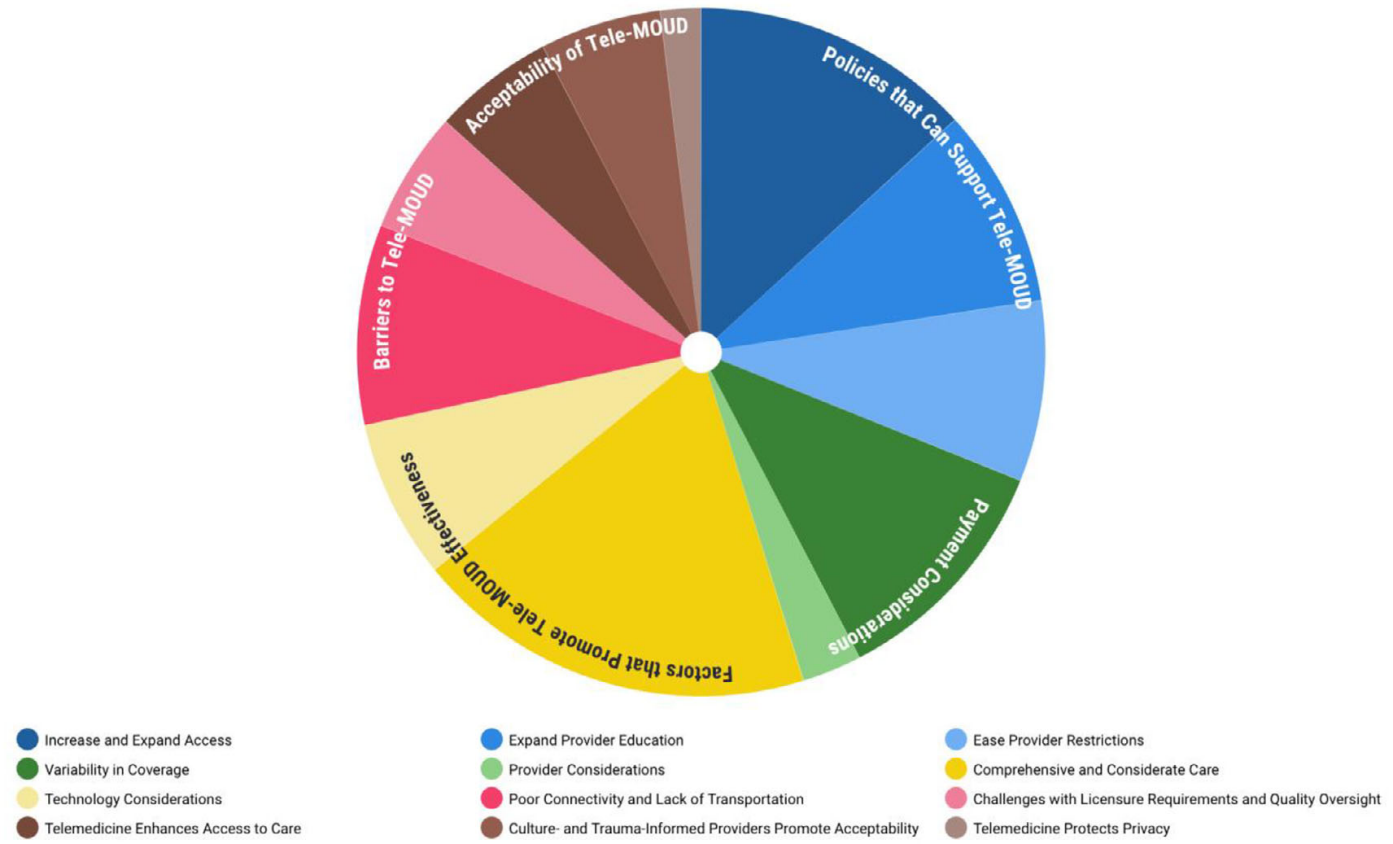
Sunburst chart summarizing the thematic areas and subcodes of provider and policy perspectives of tele‐MOUD.

The results of the coding process are displayed in Figure 1, which presents the proportion of each 6 themes that emerged: current access to tele‐MOUD, acceptability of tele‐MOUD, factors that promote tele‐MOUD, barriers to tele‐MOUD, payment considerations, and policies that support tele‐MOUD. The most prominent themes were policies that support tele‐MOUD and factors that promote tele‐MOUD effectiveness. Figure 1 also presents a breakdown of code occurrence by subcodes identified within the main themes.

### Theme 1: Current access to tele‐MOUD

Current access to tele‐MOUD was the least prominent theme in our key informant interviews, with 8 total references in 5 files. Notably, none of the respondents was aware of active tele‐MOUD programs in the Great Plains area, although they did cite familiarity with tele‐MOUD programs in the Alaska, Portland, California, Nashville, and Navajo IHS Areas.

### Theme 2: Acceptability of tele‐MOUD

This theme contained codes and statements related to whether tele‐MOUD is an acceptable form of treatment in Native communities. Statements from participants tied the acceptability of tele‐MOUD in Native communities to having culture‐ and trauma‐informed providers, especially when services are delivered by providers outside the community. When asked about the acceptability of tele‐MOUD, 1 respondent stated:

“I think if you get the right person in there, but if you just find someone who doesn't understand American Indians, who isn't culturally sensitive, who doesn't give trauma‐informed care—people who think they know and understand them, and, you know, think they're there to save them—…[patients are] not very receptive to those particular individuals.”

Partnering with telemedicine providers outside the local service unit was suggested as a means to circumvent distrust that stems from historical underfunding and poor‐quality health services for AI/AN communities. However, it was also noted that patients—especially in communities where providers have historically been transient—may see telemedicine providers as not fully committed to fostering relationships with—or ensuring favorable outcomes for—the communities served. Accordingly, some participants described the importance of in‐person visits as a supplement to tele‐MOUD to enhance the acceptability of the service.

Telemedicine was also seen as a mechanism to enhance access to care, with respondents specifically mentioning better ability to reach patients with limited transportation, decreased time requirements for patients, improved timeliness of appointments, and less infectious exposure. Pandemic‐related success in telehealth helped both providers and patients realize the value of telemedicine services, and participants maintained that demand is increasing.

The final code within the theme of acceptability incorporates statements related to how telemedicine protects privacy. In communities that are tight‐knit, substance use and mental health disorders can carry significant stigma and discourage help‐seeking. Respondents saw telemedicine as a potential solution. One respondent explained:

“For example, an Oklahoma City Area psychiatrist served people up at Pine Ridge, and they have people at Pine Ridge that they liked it because like the Oklahoma City providers kind of weren't in their business, right? They weren't connected to their family, right? And so they're like, yes, we like this!”

Another respondent added:

“[Some patients] expressed the sentiment of like ‘I don't want to tell people in the community my business’… people are so worried about their business being spread all around, particularly if they have employment…where mental health or substance abuse issues are a liability for employment, potentially.”

### Theme 3: Factors that promote tele‐MOUD effectiveness

The next theme focused on codes and statements about how to promote tele‐MOUD effectiveness. One respondent summarized it thusly:

“There's only three things that patients use to evaluate you. It's your availability, your ability, and your compatibility. If you're not available, you're of no use to the patient. If you're not compatible, the patient won't come back to see you. And if you're not competent, you will harm the patient.”

Taking each of these provider‐oriented factors individually, provider availability was cited multiple times as an important factor in promoting tele‐MOUD effectiveness. On‐call provider unavailability to patients in crisis can lead to erosion of the therapeutic relationship. “In the very fragile relationship of working with substance use disorders, that patient doesn't have faith in you anymore.” Backup support was also mentioned for times when a provider is not available or when the telehealth platform is having issues. Moreover, when the patient is in a health care setting receiving care from a remote provider, it is important to have in‐person aides available to facilitate physical exams (eg, screen for signs of withdrawal or oversedation), provide urine drug screening or other monitoring parameters, and to implement timely interventions for patients who are acutely at risk to harming themselves or others.

Multiple respondents highlighted cultural sensitivity or cultural humility as a part of compatibility, and a need to understand and respect the “very heterogeneous customs in Indian Country.” It was noted that patients “have to have a good relationship with the provider…otherwise, they don't really want to go.” Providers should avoid stigmatizing language and derogatory labeling and emphasize motivational interviewing techniques to facilitate change.

Proficiency is the final provider attribute reported for successful tele‐MOUD. Physicians need to be comfortable with the medications for MOUD, including indications, contraindications, induction dosing, maintenance dosing, and monitoring for side effects. Although the DATA Waiver was recently eliminated, 1 respondent stated that there has been little change in provider willingness to prescribe buprenorphine, stating, “Some providers are scared of doing MAT.” Our health systems must ensure that we “have providers that are competent, they understand change, they understand the substance use disorders, they understand the indication to, contraindication, and the maintenance of medications used for opioid dependence.”

Outside of provider‐related factors, participants described the physical settings in which care is delivered and comprehensive, whole‐person care. Lighting, paint color, and room layout can help patients feel more at ease. Entry into substance use care is the initial touchpoint for some patients who have avoided the health care system due to fear of stigma or judgment and presents an opportunity to provide additional services, including testing and screening for infectious diseases. The whole‐person approach was characterized this way, “So you've got to have the medical component, the behavioral health component, and hopefully, if you have a really well‐developed program, you're going to have more than that, right? You have a spiritual component.”

Participants indicated that certain technological factors must be considered. Technology was mentioned as a subtheme in both factors that promote and barriers to tele‐MOUD. Patients need to have a phone or other device capable of participating in the encounter. When using an internet‐based platform (eg, WebEx), patients need to have enough data or Wi‐Fi. They need to be technologically savvy enough to download and become familiar with the platform. Providers also need to be familiar with the platform, as well as the electronic medical record and state‐specific prescription drug monitoring program (PDMP). Some Electronic Health Records (EHRs) interface with their respective PDMPs, while others do not. Participants noted that planning for these considerations before the visit time can prevent delays in care or truncated appointment times. Providers for telemedicine will also need to be familiar and responsive to other regulations, such as cross‐state licensing or licensure compacts between states.

### Theme 4: Barriers to tele‐MOUD

The most cited barriers to tele‐MOUD were provider/community readiness, challenges with licensure requirements and quality oversight, and poor connectivity and lack of transportation. Some providers and communities have held on to the “old kind of thought” that opioid agonist therapy is dangerous or is exchanging one addiction for another. A participant explained:

“I think one of the barriers that we're seeing in implementation, I think, of MOUD in general, is site readiness, willingness, and potential stigma within the provider community. We know that there's a lot of education that still needs to occur to really, you know, enhance the provision of MOUD in general.”

Another respondent added, “We're not that far removed from when we actually fired patients because they asked for these kinds of medications, and were deemed to be drug‐seeking, right?”

It was expressed that “Licensure and delivery of telemedicine across state lines is probably one of the biggest policy issues.” Participants indicated that telemedicine providers can deliver care through contract agreements with Tribes and service units, or as credentialed and privileged members of that medical staff, but both of these arrangements take time. Contracted providers may also only be available on a rotating basis, so patients who need frequent evaluation during induction or re‐evaluation due to destabilization might not have a substance abuse provider available for several weeks.

Social determinants of health, especially related to connectivity and transportation—were the last stated barrier. Multiple respondents pointed out that patients may not have a smartphone, or may have limited minutes, data, or cellular coverage. The sunsetting of 3G cellular networks if further exacerbating coverage deserts in rural spaces. One provider acknowledged:

“30% of the patients my wife serves right now have no indoor plumbing, and no electricity, so I mean Wi‐Fi is not even on the list…I'm a firm believer in telemedicine, especially for the use for individuals who have limited access to care…But the infrastructure for telemedicine, it doesn't exist in some areas.”

Participants indicated that transportation can be cost‐prohibitive to engaging in care. Some clinics require an in‐person component, either for the telemedicine visit itself or for monitoring parameters like urine drug screening. One respondent was under the erroneous impression that controlled medicines like buprenorphine could not be mailed following a telemedicine visit. She stated:

“We do have opportunity to mail medications to patients, but not controlled substances…And so that still is a barrier for them to get it. So, you know, whether it's a CHR, who has to take it to them…or, you know, they still have to make their way to the clinic to get the medication. That that still is another barrier for them.”

### Theme 5: Payment considerations

The significant heterogeneity in the way that services are reimbursed was indicated as a consideration for implementing tele‐MOUD. Participants noted that IHS care itself is fragmented across a variety of federal, tribal (also called 638), and Urban Indian sites, with separate policies and procedures for each. Many service units rely on third‐party billing and reimbursement from Medicare, Medicaid, and private insurance to supplement their operating budget. One provider explained:

“If you look on the side of the facility, then yes, they want to make sure that their patients, you know, have some sort of insurance so they get reimbursed for some things…Because we have to rely on some sort of reimbursement in order for us to increase our services and continuing services from that perspective.”

Variability in Medicare and Medicaid funding across programs and states also complicates the picture:

“So the states are very individualized in terms of what they cover, which I think is one of the issues of complexity and one of the challenges—particularly in the Northern Plains—is that you're going to have different interpretations by Medicaid in different states. And that poses the challenge for tribes when it's not universally covered.”

Care is funded through myriad pathways and, as 1 respondent put it, “It gets very dicey.”

### Theme 6: Policies that can support tele‐MOUD

State‐licensure reciprocity or modifying the current policy for telemedicine across state lines was frequently mentioned as an area ripe for change. One provider affirmed that to increase access, “we ease any policies or restrictions that have to do with providing care across state lines, while at the same time having that visibility to oversight of quality and safety for the care that's provided.” This is especially important considering that Tribal Nation's borders may extend beyond state lines such that they are located within multiple states. Other restrictions that were mentioned were IHS‐related restriction of off‐label use of medications for substance use disorder (eg, gabapentin for alcohol use disorder or bupropion for stimulant use disorder), reimbursement for audio‐only visits, and continuation of the waiver of required in‐person visits before prescribing a controlled substance. Finally, although counseling services are an important part of caring for individuals with substance use disorders, historically high‐threshold programs (ie, programs requiring compliance with counseling and other requirements before provision of a medication) may have been a barrier for some patients, and a lower threshold program may have been preferred. One respondent cited lower threshold requirements as a form of harm reduction:

“We had really tied the actual medication with counseling and had made that kind of a standard: a lot of clinics that you had to do like so many counseling visits, to access medication…I'm a licensed clinical social worker. I think counseling is very important, but changing policies that you don't have to do that or it's not punitive.”

Respondents overwhelmingly supported the need to have increased training in and comfort with the provision of substance use care, calling for a “continued growth and development of providers in the community who are comfortable, competent, compatible, and available for this population.” Another participant suggested, “I always say, if you're an Indian country, you need to be expert at three things: one is diabetes, two is substance use disorders, and three is prevention of suicide. If you're not good at those, you don't belong in Indian country.” Respondents answered that this training can happen at the residency level or as part of professional development programs. Providers also need to be able to treat comorbid conditions, like chronic pain and depression/anxiety, to optimize outcomes.

The most prominent code within the theme of policies was policies to increase and expand services. Telemedicine relationships established during the Public Health Emergency should be allowed to perpetuate. Also, participants highlighted that, although abstinence is the goal for many patients with Substance Use Disorder (SUD), it is not the goal for all. Harm reduction services can keep people safe and allow them to seek care when ready.

## DISCUSSION

Addressing substance use disorders—especially opioid use disorder—remains a high priority for AI/AN communities. Telemedicine for the provision of MOUD appears, from this qualitative analysis, to be a promising way to expand access to care as long as technological requirements can be met. Study respondents highlighted perceived acceptability, factors promoting tele‐MOUD, barriers to implementation, and policy changes that would further facilitate change. Their responses—though specific to Great Plains Tribal Communities—mirror other recommendations on telemedicine and substance use disorder services, such as federal support of reciprocity of state licenses, permanent codification of the Public Health Emergency (PHE) regulatory changes, increased funding for innovative delivery of services, and consideration of privacy.^20^ Although IHS has made strides to ease the burden posed by state licensure requirements, many patients are also insured by Medicaid or Medicare, which have restrictions and requirements for state licensure. During the public health emergency, restrictions on practicing telemedicine across state lines were relaxed, but have now been reinstated. Of note, the DEA, together with the Department of Health and Human Services, recently issued a final rule regarding the expansion of buprenorphine treatment via telemedicine encounters.^21^

This study adds the perspective of Great Plains Tribal Communities, including the need for culturally humble and trauma‐informed providers, the stigma of receiving care for substance use disorders in a small community, and the complexities of reimbursement across Federal, Tribal, and Urban Indian Organizations. Additionally, it highlights the challenges of providing tele‐MOUD considering the intersectional experiences of rural, poverty‐stricken, geographically isolated settings with the simultaneous need to address technological challenges such as cell phones and internet connectivity. Respondents mainly focused on the physical barriers, which, although considerable and especially challenging for this population, may not be the only barriers that exist.

The disparities in IT access—the so‐called digital divide—are admittedly daunting, with almost 28% of residents residing on Tribal land lacking high‐speed internet (compared to 1.5% of urban residents).^22^ For residents who do have internet access, 50% reported limited access due to limitations in cell phone data.^23^ To combat this, former President Biden's Bipartisan Infrastructure Law and the Consolidated Appropriations Act includes the Tribal Broadband Connectivity Program, a $3 billion initiative to support Tribal governments in bringing high‐speed internet to Tribal lands.^24^ Additionally, the modernization of the IHS electronic medical record^25^ allows integrated video platforms and will enable better interfacing with the prescribing and monitoring systems necessary to provide high‐quality care. Despite existing logistical challenges, tele‐MOUD may be an approach to address the shortages of MOUD care in Great Plains Tribal Communities. This approach, coupled with additional interventions like Mobile units that bring services to patients, rather than bringing patients to services, offers an encouraging way forward. Behavioral Health Aides, now available through the IHS CHAP expansion, can be trained to help address the helplessness and hopelessness that often underpin substance use.

An important issue raised by participants was the need for prescribing clinicians who are both culturally humble and trauma‐informed. While cultural competence trainings are common, they often emphasize surface‐level knowledge of “other” cultures and may reinforce stereotypes and power imbalances.^26^, ^27^ In contrast, cultural humility emphasizes lifelong self‐reflection, recognition of bias, and openness to patients’ own expertise. This approach is particularly relevant in AI/AN communities, where adapting interventions to align with Indigenous worldviews and healing traditions may improve engagement, acceptability, and outcomes.^28^, ^29^, ^30^, ^31^ A clear example is the Swinomish Tribe's didgwálič Wellness Center, which integrated cultural perspectives into MOUD delivery and reduced overdoses among Tribal members by 50% in 1 year. Equally important, trauma‐informed care (TIC) recognizes the impact of traumatic experiences on health and highlights physical and emotional safety, trust, patient choice, and empowerment in care relationships.^32^ However, recent survey research with US physicians (N = 179) highlights that significant barriers to TIC remain, including limited time and resources, provider stress, uncertainty about appropriate referrals, and insufficient emphasis on TIC in medical education and training.^32^ Given these gaps, efforts should be made to expand physician training and organizational support for TIC, particularly when codeveloped with AI/AN communities. This may help providers meet the complex needs of patients whose social determinants of health increase their risk of trauma exposure and its lasting effects.

## LIMITATIONS

This study has several limitations that should be considered when interpreting the results. First, because this was a qualitative project, the findings are tied to the specific communities and contexts in which the work took place—that is, the Great Plains Area—and may not necessarily apply to Tribal Nations beyond those in the region. Second, while qualitative research is well‐suited to provide rich and detailed insights, it may also be influenced by researcher and interpretive bias. In this case, coding was carried out by 1 non‐Indigenous PhD student in an Indigenous Health doctorate program with extensive experience in qualitative methods. To strengthen trustworthiness, the preliminary analyses were shared with 6 additional Indigenous and non‐Indigenous team members, whose input informed the final thematic analysis. Third, given the small sample size (N = 8), the findings should be interpreted with caution, as they may not reflect the full variety of experiences within the broader provider community. However, after 5 rounds of coding, the same key themes continued to surface across the interviews, suggesting that thematic saturation had been reached. Even so, the study reflects primarily the viewpoints of individuals with professional or community involvement in substance use and related services, which are more focused on structural and service‐related issues rather than the lived realities of people currently experiencing substance use challenges. Additional exploration should include the perspectives of AI/AN individuals with lived experience of addiction and recovery to increase awareness about potential barriers and facilitators to treatment.

## CONFLICT OF INTEREST STATEMENT

The authors have no conflicts of interest to disclose.

## Supporting information



Supplement A: Semi‐structured Interview Guide
