# Supplementary material for: Exploring the use of telemedicine to expand addiction and recovery resources in Great Plains Tribal Communities
Source: J Rural Health. 2026 Jan 14;42(1):e70113. doi: 10.1111/jrh.70113 (PMC12800889; doi:10.1111/jrh.70113)
Supplement: Supplementary file 1 — Supplement A: Semi‐structured Interview Guide [file JRH-42-0-s001.docx]

**Supplement A: Semi-structured Interview Guide**

1. Is tele-MOUD currently being used for AIs in the Northern Plains or elsewhere? If so, where? Is it effective?

1. What are the barriers to providing tele-MOUD services in Northern Plains AI communities?

1. Do you think tele-MOUD is or would be an acceptable method of treatment? Why or Why not?

1. What are the payment considerations for tele-MOUD?

1. Does IHS cover or provide tele-MOUD? Directly or through PRC?

1. Does Medicaid cover tele-MOUD?

1. What is needed at the treatment site and the telemedicine site to promote effectiveness of tele-MOUD services?

1. What policies might need to change to promote use of tele-MOUD?

1. What other considerations or issues do we need to keep in mind regarding the feasibility of providing tele-MOUD in Northern Plains or other AI communities? Are there provider issues or stigma considerations?
